# In Rheumatoid Arthritis Patients, HLA-DRB1*04:01 and Rheumatoid Nodules Are Associated With ACPA to a Particular Fibrin Epitope

**DOI:** 10.3389/fimmu.2021.692041

**Published:** 2021-06-24

**Authors:** Guillaume Larid, Mikael Pancarte, Géraldine Offer, Cyril Clavel, Marielle Martin, Vincent Pradel, Isabelle Auger, Pierre Lafforgue, Jean Roudier, Guy Serre, Nathalie Balandraud

**Affiliations:** ^1^ Rhumatologie, Institut du Mouvement et de l'appareil Locomoteur (IML), Assistance Publique - Hôpitaux de Marseille (AP-HM), Marseille, France; ^2^ Université de Toulouse, INSERM, UMRs 1056, UDEAR, Hôpital Purpan, Toulouse, France; ^3^ Aix Marseille Université, INSERM UMRs 1097, Arthrites autoimmunes, Marseille, France; ^4^ CEIP de Marseille (PACA-Corse, Centre Associé), Laboratoire de Santé Publique, Faculté de Médecine, Marseille, France

**Keywords:** ACPA, rheumatoid arthritis, HLA-DRB1, AhFibA, citrullinated peptides 2

## Abstract

**Objectives:**

Rheumatoid arthritis (RA) is associated with HLA-DRB1 genes encoding the shared epitope (SE), a 5-amino acid motive. RA is usually preceded by the emergence of anti-citrullinated protein/peptide antibodies (ACPAs). Citrulline is a neutral amino acid resulting from post-translational modification of arginine involved in peptidic bounds (arginyl residue) by PeptidylArginine Deiminases (PADs). ACPAs recognize epitopes from citrullinated human fibrin(ogen) (hFib) and can be specifically detected by the AhFibA assay. Five citrullinated peptides derived from hFib together represent almost all of the epitopes recognized by patients with ACPA-positive RA, namely: α36–50cit, α171–185cit, α501–515cit, α621–635cit, and β60–74cit. The use of antibody fine specificities as markers of clinical phenotypes has become a major challenge. Our objective was to study whether RA clinical characteristics and HLA-DRB1 genetic background were associated with a specific reactivity against the epitopes borne by the five peptides.

**Methods:**

184 ACPA-positive RA patients fulfilling the 2010 ACR/EULAR criteria were studied. Patient characteristics including HLA-DRB1 genotype, were collected from their medical files. Anti-CCP2 antibodies, AhFibA, and antibodies against the five citrullinated hFib (hFib-cit) peptides were analyzed by ELISA.

**Results:**

Anti-α505-515cit antibodies were associated with HLA-DRB1*04:01 (OR = 5.52 [2.00 – 13.64]; p = 0.0003). High level anti-α505-515cit antibodies were associated with rheumatoid nodules (OR = 2.71 [1.00 – 7.16], p= 0.044).

**Conclusion:**

Immune complexes containing anti-α501-515cit antibodies and rheumatoid factors might be involved in the development of rheumatoid nodules on the HLA-DRB1*04:01 background. Apheresis of these epitope-specific antibodies might be a new therapeutic opportunity for patients with rheumatoid nodules.

## Introduction

Rheumatoid arthritis (RA) is the most severe type of chronic autoimmune arthritis. Its prevalence ranges from 0.5% to 1.1% in North America and northern Europe, and between 0.3 and 0.7% in southern Europe (1).

RA features symmetrical bilateral polyarthritis of the small joints. Extra-articular manifestations such as rheumatoid nodules, lung damage, or vasculitis can also be present (2).

RA is usually preceded by the emergence of anti-citrullinated protein/peptide antibodies (ACPAs) and rheumatoid factors of various isotypes. Citrullyl is a neutral residue resulting from post-translational modification of an arginyl residue in the peptidic sequence by PeptidylArginine Deiminases (PADs). The deiminated protein/peptide is said citrullinated.

In ACPA-positive RA, the genetic risk is mostly carried by shared epitope (SE)-positive HLA-DR molecules. The SE (a five-amino acid motif encompassing positions 70 to 74 of the HLA-DRB1 chain) encoded in the major histocompatibility complex (MHC) is present in approximately 70% of patients with ACPA-positive RA (3). Different combinations of HLA-DR alleles (genotypes) confer different relative risks of developing ACPA-positive RA, with highest risks for genotypes encoding two copies of the SE (4).

ACPA present in patients with RA recognize citrullinated epitopes on various proteins (5). A major citrullinated autoantigen expressed in the rheumatoid joint is fibrin, both its alpha and beta chain being recognized by ACPA (6). ACPA are likely to play a role in the pathophysiology of the disease. Indeed, ACPAs have been shown to predict progression of undifferentiated arthritis to RA and are associated with severe disease (7). However, given the heterogeneity of the disease’s clinical features, more reliable prognostic and phenotypic markers are missing.

The discovery of ACPA led to the development of diagnostic tests based on a first synthetic cyclic citrullinated peptides (CCP) (8). Since then, several generations of anti-CCP tests have been commercialized (9). ACPAs have become one of the 2010 American College of Rheumatology (ACR)/EULAR RA classification criteria (10).

Besides anti-CCP tests, a test for autoantibodies to human citrullinated fibrinogen (AhFibA) can be used for the serological diagnosis of early RA (11).

Five peptides from human citrullinated fibrinogen (hFib-cit) together contain almost all of the epitopes recognized by patient’s sera with ACPA-positive RA. These immunodominant epitopes are borne by the peptides α36–50cit_38,42_, α171–185cit_178,181,_ α501–515cit_510,512,_ α621–635cit_621,627,630_ and β60–74cit_60,72,74_ (6, 12, 13). Whether reactivity of sera toward these five peptides might allow definition of subgroups among RA patients that might have different disease phenotypes, is an important question.

Previous studies analyzed the recognition by various samples of patients of only 3 (α36–50cit, β60–74cit and FibCit α621-635) out of the 5 major peptides, and only studied early RA defined by the 1987 ACR criteria (14) and not the 2010 ACR/EULAR criteria.

The primary objective of this work was to study whether, in a cohort of 184 patients with ACPA-positive RA fulfilling the 2010 ACR/EULAR criteria, a particular HLA-DR background or original clinical patterns, were associated with antibodies to the epitopes from the 5 major hFib-cit peptides α36–50cit, α171–185cit, α501–515cit, α621–635cit, and β60–74cit.

## Material and Methods

### Patients

We undertook a prospective study on 184 patients followed at the rheumatology department of Sainte Marguerite Hospital in Marseille. Patients included were considered ACPA-positive RA based on previous results of anti-CCP2 antibodies obtained with various commercial assays, and fulfilled the 2010 ACR/EULAR criteria. Patients treated with Rituximab were excluded due to its potential effect on ACPA levels.

Patient characteristics were collected from their medical files: presence of rheumatoid nodules, smoking habits, age at diagnosis, HLA-DR genotype, IgM rheumatoid factor (RF), activity and erosive characteristics of the disease, treatment response, dry eye syndrome, cardiovascular event, osteoporosis.

-In order to state the presence or absence of rheumatoid nodules, patients medical files and all available chest CT scans were analyzed.
-Disease activity was measured using the Disease Activity Score calculated with the level of C-reactive protein (DAS28-CRP) on the day of the sample.
-Treatment response was evaluated according to the EULAR response criteria. Patients were considered to have shown a good response if their DAS28 was ≤3.2 or has decreased by >1.2 (15) according to the 2019 update of EULAR recommendations for the management of RA, the time needed to achieve treatment target has been set at 6 months before concluding to treatment failure (16).
-Patient personal history of a cerebrovascular accident, myocardial infarction or heart failure was defined as a major cardiovascular event.
-Patients were categorized as smokers (active or previous smokers) or non-smokers.
-Erosive RA was defined according to the EULAR definition of erosive RA (17).
-Dry eye syndrome was defined as a Schirmer's test value ≤ 5 mm/5 min, or an Ocular Staining Score ≥ 5. Dry mouth was defined as an unstimulated salivary flow rate of ≤ 0,1 mL/min.
-Osteoporosis was defined either by personal history of osteoporosis fracture, or by bone mineral density with a T-score below – 2,5 for at least one of the testing sites.

### Samples

Patients gave a single blood sample from which plasma was isolated. Samples were stored at -80°C. AhFibA and ACPA fine specificities were assayed and anti-CCP2 were re-assayed with a same test in all patients.

### Ethics

All patients gave informed written consent for this study. Patient data was pseudo-anonymized. Sample collection was approved by the ethics committee under the number DC-2008-327. This study was declared to the Assistance Publique – Hôpitaux de Marseille (AP-HM) under the number PADS19-332.

### ACPA Testing

For all the ACPA tests, we used the positivity threshold (N) corresponding to 95% diagnostic specificity.

Plasma anti-CCP2 assays were performed using the ELISA CCPlus^®^ IMMUNOSCAN kit (Euro Diagnostica, Arnhem, The Netherlands) according to the manufacturer’s recommendations. Antibody titers expressed in arbitrary units (AU/mL) were considered positive above 25 AU/mL.

Anti-human citrullinated fibrinogen IgG autoantibodies were detected with a previously described ELISA (AhFibA). Antibody “titers” (ELISA optical density) above 0.056 were considered positive (6, 18).

### Rheumatoid Factor Testing

IgM Rheumatoid Factor (RF) was assayed with the RF ELISA from Orgentec and considered positive if >20 UI/mL.

### Antibodies to Human Fibrin(ogen) Citrullinated Peptides

Peptides with the following sequences were synthetized with either arginines (R) in control peptides or citrullines (R) in target peptides, and used as immunosorbents:

-α36-50: GPRVVERHQSACKDS
-α171-185: biotin-ahx*-VDIDIKIRSCRGSCS
-α501-515: biotin-ahx*-SGIGTLDGFRHRHPD
-α621-635: RGHAKSRPVRGIHTS
-β60-74: RPAPPPISGGGYRAR

*: ahx: aminohexanoic acid

IgG autoantibodies to the fibrinogen-derived citrullinated peptides α36-50cit, α621-635cit and β60-74cit were tested by ELISA according to a previously described method in which peptides are passively adsorbed on polystyrene microtitration plates (6, 18). IgG autoantibodies to the peptides α171-185cit and α501-515cit were tested by ELISA with biotinylated peptides linked to previously adsorbed Avidin. Briefly, plates (Maxisorp; NUNC, VWR International, Fontenay-sous-Bois, France) were incubated overnight with Avidin at 5 µg/mL. After washing with PBS 0.1% Tween-20 (PBS-T) biotinylated peptides were incubated for 1 hour at 1µg/mL in PBS 2% BSA (PBS-BSA). After 3 washings the plates were incubated for 1 hour with patient plasma diluted to 1/100 in PBS-BSA then washed 3 times in PBS-T. A peroxidase-conjugated goat anti-human IgG antibody (Southern Biotechnology) diluted to 1/2500 in PBS-BSA was incubated for 1h and, after 3 washings, reactivity was revealed by ortho-phenylenediamine dihydrochloride and hydrogen peroxide in a pH5 citrate buffer. The reaction was stopped after 5 min with H_2_SO_4_ and optical density (OD) read at 492 nm. All the steps were performed at room temperature around 20°C (6). The specific antibody reactivity was defined as the OD difference between the citrullinated and the non-citrullinated related control peptide. Interassay variations were corrected by linear regression analysis using reference peptide and plasma, tested on each plate. ΔOD below the cut-off value of 0.25 were considered negative. Patients with ΔOD over the cut-off were divided into 3 equal groups (tertiles) with weak (T1), medium (T2) or high titers (T3). Patients from the medium and high-titer groups (T2-T3 combined) were considered together to have a “high level” reactivity.

### Statistical Analysis

Association studies were carried out with contingency tables using the Chi2 test and Fisher’s exact test when Chi2 was not applicable.

The Baptista-Pike method was used to obtain the 95% confidence interval for Odds Ratio (OR).

Spearman’s rank correlation coefficient rho was used to measure the correlations between anti-CCP2 antibodies, AhFibA, and antibodies to the 5 peptides and tested against the null hypothesis of rho =0.

Statistical analysis was carried out using PRISM software for Windows (GraphPad Software, 169 San Diego, California, USA) and IBM^®^SPSS^®^ Statistics version 20. The significance threshold was set at p < 0.05.

## Results

### Clinical Description of the Population

The clinical characteristics of the 184 patients with ACPA-positive RA are shown in **Table 1** (see details in **Supplementary Data File 1**). Half of patients were treated with methotrexate, 24% in monotherapy, and 76% in association with a bDMARD. Among 30 patients with rheumatoid nodules, 15/93 had methotrexate, 15/97 had not (p = 0.841, OR = 0.923 [0.44 – 1.941]).

**Table 1:** Clinical characteristics of patients.

**Number of patients**	184
**Age (years, mean)**	62
**Women (%)**	75.5
**Symptoms duration (mean, years)**	15.5
**Age at diagnosis (mean, years)**	46.4
**Rheumatoid nodules**	30/180 (16.6%)
**Smoker (active or previous)**	90/180 (50%)
**DAS28 at inclusion**	2.44
**Major cardiovascular event**	15/180 (8.3%)
**Erosive rheumatoid arthritis**	64/180 (35.5%)
**Dry eye syndrome**	11/167 (6.6%)
**Rheumatoid Factor**	121/161 (75%)
**Methotrexate treated**	96/184 (52.2%)
**Monotherapy**	23/96 (24.0%)
**Associated with other DMARDs**	73/96 (76.0%)
**Treatment**	**Good responders/number treated**
**TNF-alpha inhibitors**	90/137 (65.6%)
**Tocilizumab**	37/53 (69.8%)
**Abatacept**	24/46 (52.1%)

### Serological Profile of the 184 Patients With ACPA-Positive RA

The biological characteristics of the 184 patients recruited as ACPA-positive RA based on their anti-CCP2 status are shown in **Table 2** (see details in **Supplementary Data File 1**). When re-assayed with the same reference assay, 98.51% of patients had anti-CCP2 antibodies. On the other hand, 83.23% had AhFibA, and 69.98% had IgM RF.

**Table 2:** AhFibA and five major citrullinated Fibrin peptides antibodies.

	Negative	Positive
AhFibA	27/184 (14.67%)	157/184 (85.33%)
β60–74cit	76/184 (41.30%)	108/184 (58.70%)
α36–50cit	157/184 (85.33%)	27/184 (14.67%)
α621–635cit	103/184 (55.98%)	81/184 (44.02%)
α501–515cit	62/184 (33.70%)	122/184 (66.3%)
α171–185cit	45/184 (24.46%)	139/184 (75.54%)

#### Among the Citrullinated Peptides

Among the citrullinated peptides, 76.13% of patients recognized biotin-α171-185cit, 67.10% recognized biotin-α501-515cit, 58.06% recognized β60-74cit, 45.16% recognized α621-635cit, and 14.84% recognized α36-50cit.

#### Epitopic Profiles of ACPA

**Figure 1:**
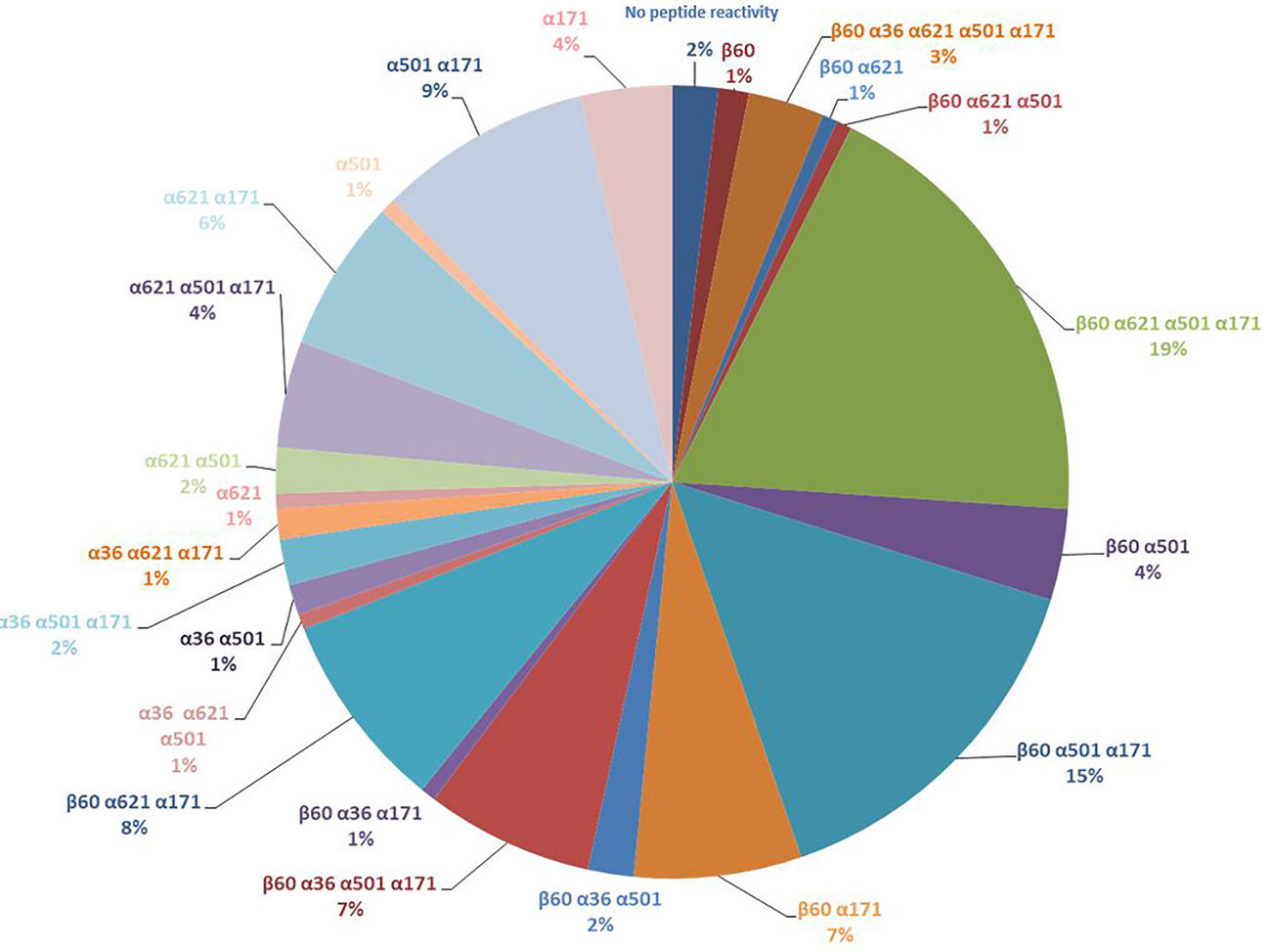
Different profiles of epitopic reactivity in the cohort of 184 patients.

Analysis of the profiles of epitope recognition in the 184 patients showed that 19% of patients recognized the 4 peptides α171-185cit, α501-515cit, α621-635cit, and β60-74cit, 15% of patients recognized the 3 peptides α171-185cit, α501-515cit, and β60-74cit, and 6% of patients recognized a single peptide (4%: α171-185cit, 1%: α501–515cit, and 1%: β60–74cit) (see **Figure 1**).

### Correlations of the Titers of Anti-CCP2 and Anti-Citrullinated Fibrin Peptide Antibodies

The titers of anti-CCP2 and AhFibA were strongly correlated (Spearman’s rank correlation coefficient rho (rs): 0.706; p= 4.06x10^-29^).

The titers of anti-CCP2 and anti-citrullinated fibrin peptides antibodies were also significantly correlated for 4 of the 5 peptides: α171-185cit (rs: 0.647; p = 3.2x10^-23^), α501-515cit (rs: 0.299; p = 3.7x10^-5^), α621-635cit (rs: 0.271; p= 0.0002), β60-74cit (rs: 0.517; p = 5.5x10^-14^).

**Figure 2:**
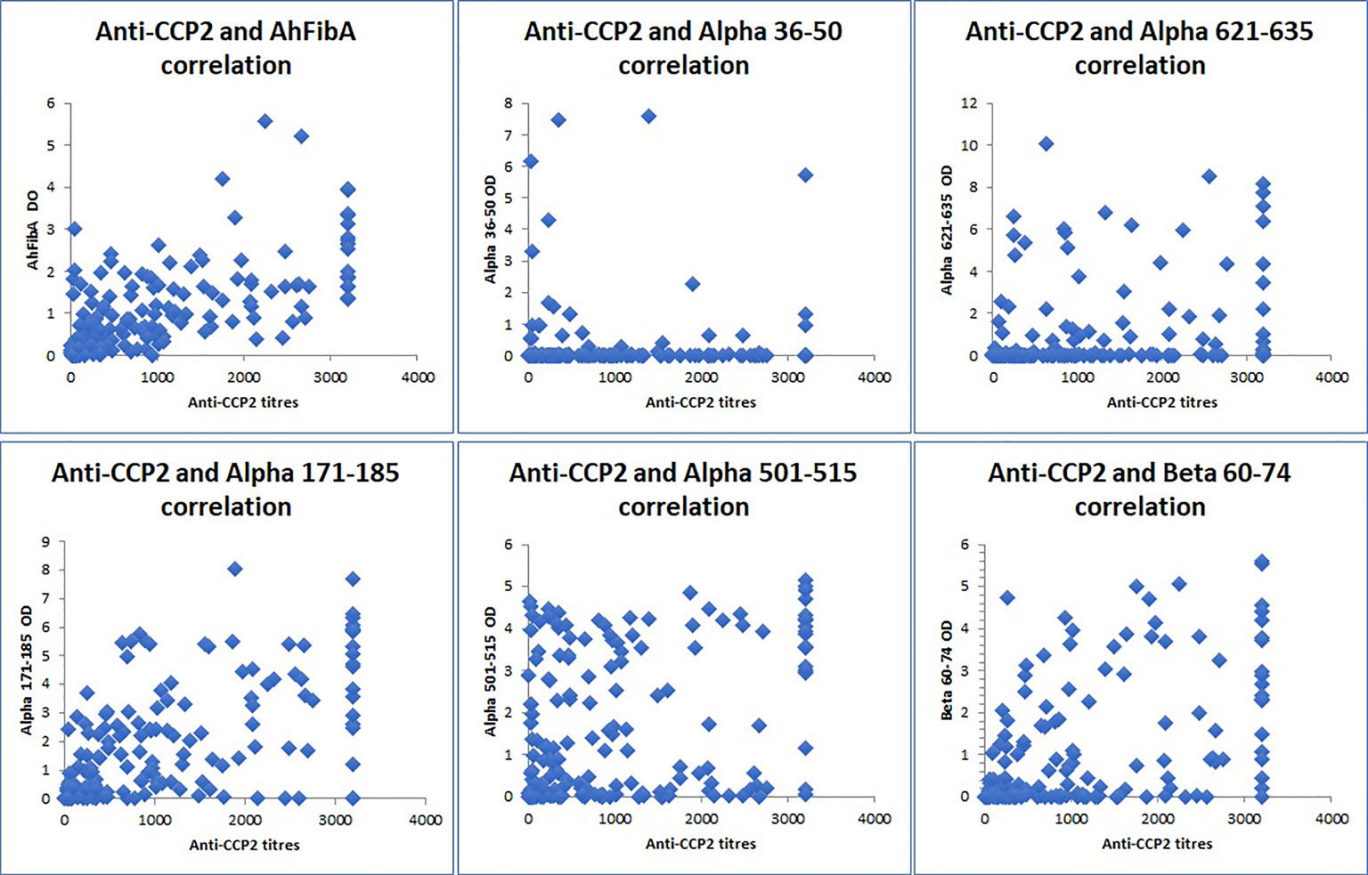
Correlations of anti-CCP2 titers and anti-citrullinated fibrin peptides antibodies (Spearman’s rank correlation coefficient rho (rs). OD , optical density; AhFibA , anti-citrullinated human fibrinogen antibodies; anti-CCP2, anti-cyclic citrullinated peptides. There was a positive correlation between anti-CCP2 and AhFibA (r_s_ : 0.706; p = 4.06x10^-29^), between anti-CCP2 and anti-α171-185cit (rs: 0.647; p = 3.2x10^-23^), anti-α501-515cit (rs: 0.299; p = 3.7x10^-5^),anti-α621-635cit (rs: 0.271; p= 0.0002), and anti-β60-74cit antibodies (rs: 0.517; p = 5.5x10^-14^). There was no correlation between anti-CCP2 and anti α36–50 (rs: -0.010; p = 0.894) antibodies.

By contrast there was no correlation of anti-CCP2 antibodies titers with those of anti-α36-50cit antibodies (rs: -0.010; p = 0.894) (see **Figure 2**).

### HLA-DRB1 Background

Among the 184 patients with ACPA-positive RA, 155 were genotyped for HLA-DRB1: 67.1% of patients were SE-positive (49% of patients expressed one copy of the SE, 18.1% expressed two copies). Their clinical characteristics were not different from those in the total 184 patients (see **Supplementary Tables 1**, **2**, **Supplementary Data File 1**).

#### HLA-DR Status Association With Clinical Characteristics

HLA-DRB1*04:01 was weakly associated with rheumatoid nodules. Indeed, 44/155 patients expressed HLA-DRB1*04:01 of whom 11 (25%) had rheumatoid nodules, 111/155 patients did not express HLA-DRB1*04:01 of whom 19 (17%) had rheumatoid nodules (Chi2, OR = 2.15 [0.91 – 5.25]; p = 0.081). Other clinical characteristics were not associated with any HLA-DRB1 allele.

#### HLA-DRB1 Status Association With the Recognition of Citrullinated Peptides

##### SE Dose Effect on the Number of Peptides Recognized

Patients with two copies of the SE were more likely to recognize more than 3 peptides (OR = 3.02 [1.12 – 8.74]; p = 0.034) (see **Table 3**).

**Table 3:** Association between epitopic specificity of ACPAs and HLA-DRB1 alleles.

		Shared Epitope				HLA-DRB1*04:01				HLADRB1*04:04,*04:05,				HLADRB1*10:01			
		N=104/155				N=44/155				*04:08,*01 N=69/155				N=14/155			
		NO	YES	Odds Ratio	p	NO	YES	Odds Ratio	p	NO	YES	Odds Ratio	p	NO	YES	Odds Ratio	p
		(n)	(n)	[CI 95%]		(n)	(n)	[CI 95%]		(n)	(n)	[CI 95%]		(n)	(n)	[CI 95%]	
**β60-74cit**	NO (n)	25	40	1.54	0.21	49	16	1.38	0.37	37	28	1.11	0.76	61	4	1.91	0.29
				[0.80 – 2.98]				[0.68 – 2.84]				[0.58 – 2.13]				[0.60 – 5.72]	
	YES (n)	26	64			62	28			49	41			80	10		
**α36-50cit**	NO (n)	45	88	1.36	0.54	95	38	0.93	0.90	77	56	1.99	0.14	120	13	0.44	0.42
				[0.54 – 3.68]				[0.34 – 2.41]				[0.804 – 4.69]				[0.04 – 2.68]	
	YES (n)	6	16			16	6			9	13			21	1		
**α621-635cit**	NO (n)	23	62	0.55	0.08	55	30	0.46	**0.04**	45	40	0.80	0.48	79	6	1.70	0.34
				[0.28 – 1.06]				[0.23 – 0.95]				[0.42 – 1.50]				[0.53 – 5.27]	
	YES (n)	28	42			56	14			41	29			62	8		
**α501–515cit**	NO (n)	23	28	2.23	**0.02**	46	5	5.52	**0.0003**	31	20	1.38	0.35	45	6	0.63	0.40
				[1.10 – 4.55]				[2.00 – 13.64]				[0.71 – 2.77]				[0.19 – 1.97]	
	YES (n)	28	76			65	39			55	49			96	8		
**α171–185cit**	NO (n)	15	22	1.55	0.26	30	7	1.96	0.14	22	15	1.24	0.58	35	2	1.65	0.52
				[0.74 – 3.28]				[0.78 – 5.00]				[0.60 – 2.71]				[0.41 – 7.81]	
	YES (n)	36	82			81	37			64	54			106	10		
**Number of specificities**	≤3	42	77	1.63	0.25	85	34	0.96	0.92	69	50	1.54	0.25	111	8	2.78	0.07
				[0.71 – 4.01]				[0.42 – 2.20]				[0.74 – 3.28]				[0.85 – 8.31]	
	>3	9	27			26	10			17	19			30	6		

N, number. Bold values are the significant ones.

##### HLA-DRB1 Alleles and Epitopic Specificity

HLA-DRB1*04:01 was associated with antibodies to α501-515 (OR = 5.52 [2.00 – 13.64]; p = 3x10^-4^). Conversely, HLA-DRB1*04:01 was associated with absence of antibody to α621-625 (OR=0.46 [0.23 – 0.95]; p=0.036) (see **Table 3**).

### Epitopic Specificity of Anti-Citrullinated Fibrin Peptide Antibodies and Clinical Characteristics

#### Multiple Peptide Recognition

42 of 184 patients recognized more than 3 citrullinated peptides. The 142 remaining patients recognized 3 peptides or less. Patients who recognized more than 3 peptides showed better responses to TNF-alpha inhibitors (OR = 2.93 [1.16 – 7.48]; p = 0.025) and had less often erosive RA (OR = 0.45 [0.19 – 1.04]; p = 0.051) (see **Supplementary Table 3**).

#### Recognition of the α501-515 Citrullinated Peptide Was Associated With Rheumatoid Nodules and With RF

Rheumatoid nodules were present in 30/180 patients, absent in 130 patients, data being unknown for 20 patients. On the whole group of patients, reactivity against α501-515cit tended to be associated with rheumatoid nodules (OR= 2.32 [0.95 -5.80], p = 0.08) but high level reactivity against the peptide was significantly associated with rheumatoid nodules (see **Table 4**). Indeed, 79/184 (43%) patients had high level reactivity against α501-515cit and among them 18/184 (10%) patients had rheumatoid nodules whereas 61/184 (33%) patients had low level reactivity against α501-515cit of whom only 6/184 (3%) patients had rheumatoid nodules (OR=2.71 [1.00 – 7.16], p=0.044).

**Table 4:** Rheumatoid nodules and Rheumatoid factor association with peptide reactivity.

		Rheumatoid Nodules				Rheumatoid Factor			
		(n = 30/180)				(n = 122/161)			
		NO	YES	Odds Ratio	p	NO	YES	Odds Ratio	p
		(n)	(n)	[CI 95%]		(n)	(n)	[CI 95%]	
**Rheumatoid Factor** **(n=122/161)**	NO	35	3	**2.89 [0.88 – 9.52]**	**0.08**				
	(n)								
	YES	97	24						
	(n)								
**β60-74cit**	NO	63	11	1.25 [0.55 – 2.69]	0.59	15	54	0.79 [0.37 – 1.68]	0.52
	(n)								
	YES	87	19			24	68		
	(n)								
**α36-50cit**	NO	130	26	0.23 [0.33 – 2.89]	0.99	36	102	2.35 [0.70 – 7.83]	0.17
	(n)								
	YES	20	4			3	20		
	(n)								
**α621-635cit**	NO	81	19	0.68 [0.32 – 1.53]	0.35	25	68	1.42 [0.66 – 2.88]	0.36
	(n)								
	YES	69	11			14	54		
	(n)								
α501–515cit**	NO	55	6	**2.32 [0.95 – 5.80]**	0.08**	18	33	2.31 [1.10 – 4.78]	**0.026**
	(n)								
	YES	95	24			21	89		
	(n)								
**α171–185cit**	NO	37	7	1.08 [0.45 – 2.75]	0.88	11	30	1.21 [0.56 – 2.68]	0.65
	(n)								
	YES	113	23			28	92		
	(n)								
**Number of specificity**	≤ 3	119	21	1.65 [0.69 – 3.78]	0.26	35	93	2.73 [0.91 – 7.61]	**0.07**
	> 3	31	9			4	29		

N, number of patients; NS, not significant.

**High level anti-α501–515cit (T2-T3 tertiles combined) was significantly associated with rheumatoid nodules, **OR =2.71 [1.00 – 7.16], p= 0.044.** 79/184 patients had high level anti- α501–515cit, among them 18 patients had rheumatoid nodules, 61/184 patients did not have high level anti- α501–515cit, of whom 6 patients had rheumatoid nodules. Bold values are the one which are important regarding the text of the article.

Anti-α501-515cit antibodies were also associated with RF (OR=2.31 [1.10 – 4.78], p= 0.026).

## Discussion

We studied the associations between HLA-DR background, specificity of ACPAs to epitopes borne by citrullinated fibrin peptides and clinical characteristics in 184 patients with ACPA-positive RA fulfilling the ACR/EULAR 2010 criteria.

### The Clinical and Biological Characteristics

The clinical and biological characteristics of the RA population studied here were classical. In our population of patients with ACPA-positive RA, 17% of patients had rheumatoid nodules which is in accordance with other European populations prevalence of 18%-22.4% and less than in northern European population (30-40%) (19). It was previously reported that cvDMARDs or bDMARDs could accelerate rheumatoid nodulosis, particularly methotrexate (20) and TNF-alpha inhibitors (21). In our cohort we could not find any over-representation of rheumatoid nodules in patients treated with DMARDs, specially with methotrexate.

### Prevalence of ACPAs to the Citrullinated Fibrin Peptides

The prevalence of antibodies to the peptides α36-50cit (15%), α621-635cit (45%), and β60-74cit (58%), were in close agreement with those previously described with the same ELISAs (11, 13, 22–26) and also those recently described in very large cohorts of patients with a multiplex peptide chip (27–32). The pilot study (6) which identified the peptides bearing the immunodominant epitopes, only used sera selected for highest titers of ACPA, therefore leading to prevalences which are not representative of patient population.

The prevalence of antibodies to the peptides α171-185cit (76%) and α501-515cit (67%) had been previously studied by ELISA only in the pilot study (6) and were 45% for the 2 peptides. However in the present study, the peptides were used biotinylated and linked to Avidin versus immunoadsorbed on solid phase in the pilot study. Such an increased reactivity when using biotinylated peptides, depending on a different presentation of the peptides, was clearly demonstrated with the peptide β60-74cit in a recently published study (33).

### Association Between Multiple Peptide Recognition and Clinical Characteristics

Multiple peptide recognition (>3) was not associated with erosive arthritis, in accordance with previous studies that showed that the diversity of recognized epitopes was not associated with the severity of the disease (34, 35). Multiple peptide recognition (>3) was associated with a better response to TNF-alpha inhibitors in our cohort. In the literature, ACPA have been associated either with a reduced response (36), no effect (37) or a better response (38). A better response to TNF-alpha inhibitors may explain the less erosive pattern observed in our patients with multiple peptide recognition. Indeed, TNF-alpha inhibitors limit radiographic progression in patients with RA as already demonstrated (39).

A study of van Beers et al. (40) failed to find association between ACPA fine-specificity profiles in early rheumatoid arthritis patients and clinical features at baseline or with disease progression, however, the citrullinated peptides used in our study were not analyzed, neither rheumatoid nodules.

Recently, Manca et al. studied the clinico-serological association of ACPA detected using VCP1 and VCP2 (EBV-derived citrullinated peptides) and HCP1 and HCP2 (histone-H4-derived citrullinated peptides) in 413 established RA. Higher number/levels of ACPA subtypes were associated with lung involvement but not with erosive disease (41).

In Joshua and al. association between ACPA fine specificities and lung abnormalities were studied. Three out of our 5 antibodies (FibCit α36-50, FibCit β60-74, and FibCit α621-635) were used in the study. There was no association between reactivity to any of them and lung abnormalities studied by HR-CT scan but in contrast a significant correlation when reactivity to the various fibrin peptides was considered as a whole (42).

Here, CT scanners were not available for the whole population studied, so we could not confirm any association between number of specificities and lung involvement.

### Association Between Particular Anti-Citrullinated Fibrin Peptide Antibodies, HLA Background, and Clinical Characteristics

We found that: 1/HLA-DRB1*04:01 was associated with anti-α501-515cit antibodies, 2/Rheumatoid nodules were associated with HLA-DRB1*04:01, 3/”High level” anti-α501–515cit antibodies were associated with rheumatoid nodules and with IgM rheumatoid factor.

#### HLA-DRB1*04:01 Was Associated With Anti-α501–515cit Antibodies

Associations between fine-specificity antibodies and the presence of the SE have been already analyzed.

Pratesi et al. studied viral citrullinated peptides (VCP) derived from Epstein-Barr virus nuclear proteins EBNA-1 and EBNA-2. Analyzing the contribution of individual SE alleles, the *0401 allele conferred an increased risk to produce anti-VCP2 (p = 0.007) and anti-CCP2 (p = 0.02) antibodies whereas *0404 was associated with the production of anti-VCP1 (p = 0.05) and anti-VCP2 (p = 0.04) antibodies (43).

The C. Terao et al. study (29) investigated whether subtle differences in ACPA serological profiles might be connected to different driving HLA alleles, by analyzing 18 ACPA fine-specificities in RA-affected individuals. A clustering of the peptide reactivities led to divide the patients as having “non-canonical” (including CCP2-negative) or “canonical” serologies (including CCP2-positive). When associations with HLA-A, B, and DR alleles were analyzed, a significant association with aspartic acid residue at Bpos-9 was found between patients with non canonical serologies and an inverse correlation with the canonical ones. In the multiplex peptide array used in that study, 7 peptides from Fibrinogen were present among the 18 tested but only 3 of our 5 peptides, namely α36–50cit, α621–635cit, and β60–74cit. Thus, our study appears as the first correlation analysis of HLA-DR and reactivities to α171–185cit and α501–515cit.

Here, HLA-DRB1*04:01 was associated with anti-α501–515cit antibodies.

In the development of IgG antibody responses, HLA-DR molecules intervene by presenting antigenic peptides to T follicular helper (Tfh) lymphocytes that provide help to B lymphocytes with surface IgM of a given specificity. Specificity of the antibody response relies on the IgM molecule on the B lymphocyte, not on the antigenic peptide presented by-HLA-DR. Thus, association of HLA-DRB1*04:01 with anti-α501-515cit antibodies does not indicate that this RA-associated allele is capable to choose between different B cell epitopes. Rather it suggests that HLA-DRB1*04:01 can provide help to B lymphocytes specific for the α501-515cit peptide and that others HLA-DR alleles can’t. This suggests special efficacy of HLA-DRB1*04:01 as a peptide processor and presentator which in turn allows the development of a more efficient Tfh response. HLA-DR molecules encoded by HLA-DRB1*04:01 have been shown to undergo a shortcut from endoplasmic reticulum to lysosomes which allows association with better processed antigenic peptide (44, 45).

#### HLA-DRB1*04:01 Was Associated With Rheumatoid Nodules

Association of HLA-DRB1*04:01 with rheumatoid nodules is classical. More, in a studied population, rheumatoid nodule frequency correlates directly with the prevalence of HLA-DRB1*04:01 of this population (19).

#### “High Level” Anti-α501-515cit Antibodies Were Associated With Rheumatoid Nodules and With IgM Rheumatoid Factor

Association between high level anti-α501-515cit antibodies, RF and rheumatoid nodules refers to rheumatoid nodules nature. As previously described, they contain fibrin and IgG but nothing is known about the specificity of those IgG. Necrotic centers were shown to contain citrullinated proteins and RF (46, 47). Our data suggests that at least some of the IgG are anti-α501-515cit antibodies. They might form immune complexes with citrullinated fibrin secondarily transformed in macroimmune complexes by rheumatoid factors (48, 49) and be at the origin of rheumatoid nodules.

That link of reactivity to a particular fibrinogen peptide and the presence of rheumatoid nodules needs to be further examined and replication studies must be performed. Moreover, while these findings raise new hypothesis about the pathogenesis of rheumatoid nodules, no study of antigen specificity of the IgG present in the nodules has been performed to date. It could be the purpose of further investigations.

In this cohort we analyzed the epitopic specificity of ACPAs using five major citrullinated fibrin peptides which bear the immunodominant ACPA epitopes and checked whether the specificity was associated with particular clinical, biological, or genetic patterns. Our main finding is the association of antibodies to the epitope borne by the citrullinated peptide α501–515 on the alpha chain of fibrin, with HLA-DRB1*04:01 and with rheumatoid nodules. This unexpected epitope specificity of ACPAs in HLA-DRB1*04:01 patients may relate to the original intracellular route of HLA-DRB1*04:01. The association of anti-α501-515cit antibodies with rheumatoid nodules may suggest that the antibodies to the epitope borne by the α501-515cit peptide particularly contribute to the immune complexes in rheumatoid nodules. This finding pave the way of a potential treatment of rheumatoid nodules by purifying patient’s serum from this particular ACPA subtype.

## Data Availability Statement

The raw data supporting the conclusions of this article will be made available by the authors, without undue reservation.

## Ethics Statement

The studies involving human participants were reviewed and approved by Comité d’éthique Aix Marseille Université - DC-2008-327. The patients/participants provided their written informed consent to participate in this study.

## Author Contributions

NB, GS, and GL designed research and analyzed data. MM, NB, and IA collected samples. MP, GO, and CC tested and analyzed fine specificities. VP performed statistical analysis. GL, NB, and PL analyzed patients files. GL, NB, and JR wrote the manuscript. NB, JR, and GS corrected the manuscript. All authors contributed to the article and approved the submitted version.

## Funding

This study was supported by INSERM. 

## Conflict of Interest

The authors declare that the research was conducted in the absence of any commercial or financial relationships that could be construed as a potential conflict of interest.
