# Supplementary material for: In Rheumatoid Arthritis Patients, HLA-DRB1*04:01 and Rheumatoid Nodules Are Associated With ACPA to a Particular Fibrin Epitope
Source: Front Immunol. 2021 Jun 24;12:692041. doi: 10.3389/fimmu.2021.692041 (PMC8264359; doi:10.3389/fimmu.2021.692041)
Supplement: Supplementary file 2 [file Table_1.docx]

**Supplementary Table 1:** Clinical characteristics of the HLA-DRB1 genotyped population

| Number of patients | 155 |
| --- | --- |
| Age (years, mean) | 61.781 |
| Women (%) | 75.817 |
| Diagnostic delay (mean, years) | 1.013 |
| Age at diagnosis (mean, years) | 46.68 |
| Rheumatoid nodules | 26/152 (17.11 %) |
| Ever smoker | 78/153 (50.98 %) |
| DAS28 at inclusion | 2.49 |
| Major cardiovascular event | 15/152 (9.87 %) |
| Erosive rheumatoid arthritis | 52/152 (34.21 %) |
| Mean number of erosions | 5.357 |
| Osteoporosis | 28/146 (19.18 %) |
| Dry eye syndrom | 11/150 (7.33 %) |
| treatment | good responders/ number treated |
| TNF alpha inhibitors | 80/117 (68.4%) |
| Tocilizumab | 27/41 (65.8%) |
| Abatacept good | 23/39 (58.9%) |
