# Supplementary material for: In Rheumatoid Arthritis Patients, HLA-DRB1*04:01 and Rheumatoid Nodules Are Associated With ACPA to a Particular Fibrin Epitope
Source: Front Immunol. 2021 Jun 24;12:692041. doi: 10.3389/fimmu.2021.692041 (PMC8264359; doi:10.3389/fimmu.2021.692041)
Supplement: Supplementary file 3 [file Table_2.docx]

**Supplementary Table 2 :** Biological characteristics and serological profile of the genotyped population

|  | Negative | Positive  Positifs |
| --- | --- | --- |
| AhFibA | 26/155 (16.77 %) | 129/155 (83.23 %) |
| β60–74cit | 65/155 (41.94 %) | 90/155 (58.06 %) |
| α36–50cit | 132/155 (85.16 %) | 23/155 (14.84 %) |
| α621–635cit | 85/155 (54.84 %) | 70/155 (45.16 %) |
| α501–515cit | 51/155 (32.90 %) | 104/155 (67.10 %) |
| α171–185cit | 37/155 (23.87 %) | 118/155 (76.13 %) |
