# Supplementary material for: In Rheumatoid Arthritis Patients, HLA-DRB1*04:01 and Rheumatoid Nodules Are Associated With ACPA to a Particular Fibrin Epitope
Source: Front Immunol. 2021 Jun 24;12:692041. doi: 10.3389/fimmu.2021.692041 (PMC8264359; doi:10.3389/fimmu.2021.692041)
Supplement: Supplementary file 4 [file Table_3.docx]

**Supplementary Table 3:** Associations between clinical characteristics and ACPA epitopic specificities

|  | **TNF-alpha inhibitors: good responders (n = 90/137)** | | **Tocilizumab: good responders**  **(n =37/ 53)** | | **Abatacept: good responders**  **(n = 24/46)** | | **Erosive rheumatoid arthritis**  **(n = 64/180)** | | **Smoker (active or previous)**  **(n = 90/180)** | |
| --- | --- | --- | --- | --- | --- | --- | --- | --- | --- | --- |
|  | Odds Ratio  [CI 95%] | p | Odds Ratio  [CI 95%] | p | Odds Ratio  [CI 95%] | p | Odds Ratio  [CI 95%] | p | Odds Ratio  [CI 95%] | p |
| **Rheumatoid factors**  **(n= 121/161)** | 1.13 [0.49 – 2.70] | ns | 1.28 [0.35 – 4.81] | ns | 0.66 [0.20 – 2.33] | ns | 0.91 [0.44 – 1.87] | ns | **2.37 [1.10 – 4.78]** | **0.022** |
| **Positivity β60-74cit** | 1.22 [0.61 – 2.55] | ns | 0.79 [0.23 – 2.53] | ns | 0.68 [0.22 – 2.39] | ns | 1.03 [0.57 – 1.91] | ns | 1.45 [0.80 – 2.67] | ns |
| **Positivity α36-50cit** | 1.96 [0.72 – 5.10] | ns | 0.53 [0.13 – 2.34] | ns | 4.20 [0.58 – 53.48] | ns | 0.67 [0.26 – 1.66] | ns | 0.58 [0.25 – 1.31] | ns |
| **Positivity α621-635cit** | 1.54 [0.74 – 3.18] | ns | 0.98 [0.33 – 3.15] | ns | **2.92 [0.84 – 9.82]** | **0.077** | 0.71 [0.38 – 1.35] | ns | 1.26 [0.70 – 2.27] | ns |
| **Positivity α501–515cit** | 1.03 [0.48 – 2.13] | ns | **0.23 [0.05 – 1.07]** | **0.065** | 1.43 [0.41 – 5.38] | ns | 0.97 [0.51 – 1.79] | ns | 1.16 [0.63 – 2.15] | ns |
| **Positivity α171–185cit** | 0.89 [0.38 – 2.09] | ns | 1.21 [0.35 – 4.86] | ns | 0.59 [0.17 – 2.15] | ns | 1.37 [0.66 – 2.77] | ns | 1.44 [0.74 – 2.91] | ns |
| **Number of positivities** | **2.93 [1.16 – 7.48]** | **0.025** | 0.61 [0.16 – 2.16] | ns | **4.12 [0.90 – 21.06**] | **0.086** | **0.45 [0.19 – 1.04]** | **0.051** | 1.56 [0.78 – 3.08] | ns |
